# The Role of Vitamin D in Hematologic Disease and Stem Cell Transplantation

**DOI:** 10.3390/nu5062206

**Published:** 2013-06-18

**Authors:** Aric C. Hall, Mark B. Juckett

**Affiliations:** Division of Hematology and Medical Oncology, School of Medicine and Public Health, University of Wisconsin, Madison, WI 53705, USA; E-Mail: mbj@medicine.wisc.edu

**Keywords:** vitamin D, differentiation therapy, acute leukemia, allogeneic stem cell transplant

## Abstract

Vitamin D is a steroid hormone with a broad range of biological effects ranging from the classical role as a mediator of calcium and phosphate balance to cellular differentiation and immune modulation. These effects impact normal and dysfunctional hematopoietic and immune function, which may allow an avenue for improved treatment and support of patients suffering from hematologic disorders. In this review, we will summarize the role of vitamin D in normal hematopoiesis, discuss ways in which vitamin D may improve outcomes, and discuss a potential role of vitamin D for treating hematologic disorders and modulating the immune system to improve the outcome of allogeneic stem cell transplant.

## 1. Introduction

Vitamin D is a steroid hormone whose classical role is as a regulator of calcium and phosphate metabolism. This action, carried out through the renal, skeletal, and gastrointestinal systems has accumulated additional attention over the last several years with osteoporosis and bone health becoming an increasingly important concern for an aging population. Further investigation has discovered high rates of vitamin D deficiency in the United States population leading to a proliferation of recommendations for supplementation and investigation into the activity of the hormone. As investigators pursued the biochemical targets of vitamin D that influenced calcium homeostasis, multiple other roles of vitamin D signaling were discovered in various tissues that express vitamin D receptors (VDRs). VDRs have been identified on hematopoietic and lymphoid cells, leading to efforts to understand the role of vitamin D in blood cell development and immune system function. In this review we will attempt to give an overview of the role of vitamin D in normal hematopoietic and immune function, explore experience at exploiting vitamin D receptor signaling to treat hematologic disorders, discuss roles of the vitamin D receptor in immune recovery following hematopoietic stem cell transplant, and finally discuss the use of vitamin D in hematology and stem cell transplant patients.

## 2. Vitamin D in Normal Hematopoiesis

### 2.1. Vitamin D Production and Mechanism of Biological Action

Human vitamin D production requires the skin, liver, and kidney to convert 7-dehydrocholesterol in the skin to 1,25-dihydroxycholecalciferol, the active form of the hormone. Dehydroxycholesterol is converted to cholecalciferol (Vitamin D3) with UV light as a cofactor and then goes through two hydroxylation steps to 25-hydroxycholecalciferol and then 1,25-dihydroxycholecalciferol in the liver and kidney [1]. Vitamin D2 (ergocalciferol) is produced in some fungi and invertebrates in an analogous manner, as ergosterol is converted to ergocalciferol with UVB light as a necessary cofactor [2]. Ergocalciferol can be absorbed from the intestines when taken as a supplement and is similarly hydroxylated for activity so that, like 1,25 dihydroxyvitamin D, it can activate the vitamin D receptor (VDR). Vitamins D2 and D3 can both be referred to as vitamin D and references differ as to their biological equivalence. In general, authors who contend a difference in biological activity suggest vitamin D3 is more biologically potent [3]. The VDR is the mediator of vitamin D activity. It is a receptor in the family of steroid/thyroid hormone-activated transcription factors. The gene for VDR is encoded on chromosome 12, and is subject to significant variability among individuals due to numerous polymorphisms [4]. Several germ line variants of the vitamin D receptor gene (alleles) are known to exist, explaining at least part of the variability in VDR activity among individuals [5]. These polymorphisms have been linked to such diverse biological effects as adult height, bone mineral density, and susceptibility to early periodontal disease and tuberculosis [6,7].

After being bound by activated vitamin D, VDR can form a homodimer or form a heterodimer with a retinoid X receptor. Those dimers can then binds to vitamin D response elements (VDREs) in promoters of target genes eventually leading to target gene transcription [1,4]. Screens for VDREs have revealed them in multiple growth, differentiation, and apoptosis governing genes including some known to be involved in human malignancy such as cyclin D1; Cyclin dependent kinases 2,4,6; PTEN; P53; and PI3 Kinase [8]. 

### 2.2. Vitamin D Role in Myeloid Differentiation

Aside from its ubiquity in affecting the transcription of many genes, VDR is present on a wide variety of tissues outside the intestine, bones, and kidneys, which are the organs most involved in the classical role of vitamin D. In the hematopoietic system the VDR receptor is expressed on various hematopoietic precursors as well as monocytes, some thymocytes, and activated B and T lymphocytes [8]. Studies with knock-out (KO) animals show vitamin D signaling does not appear to be necessary for differentiation of the normal hematopoietic cell repertoire, as mice with vitamin D receptor knock-out produce normal numbers and proportions of blood cells [8,9,10]. Still, vitamin D stimulation can influence hematopoietic development as experiments treating both normal hematopoietic stem cell lines and leukemic cell lines with the active form of vitamin D led to increased monocyte/macrophage differentiation and increased numbers of those mature cells–an effect that is not observed in VDR KO mice [11,12]. After being bound by a vitamin D analogue, the VDR forms a homodimer or binds the retinoid X receptor and proceeds to interact with a VDRE and signal transcription of various effector RNAs [9,11]. The retinoic acid receptor (RAR) is an alternative dimerization partner for the retinoid X receptor [13]. Just as VDR activity seems to drive monocytic differentiation, the RAR activity drives differentiation toward mature granulocytes [11]. Experiments in cell cultures suggest that activated RAR and VDR compete for binding of RXR and the relative balance between RAR/RXR *vs.* VDR/RXR dimers influence the relative activity of monopoiesis and granulopoiesis [11,14]. A double knock-out animal for VDR and RAR create appropriate colony forming units for monocytes and granulocytes so it is suspected that the primary effect of vitamin D is on cytokine signaling and the final steps in differentiation in these two cell types [11]. The complexity of the interaction between VDR and RXR is highlighted by a recent finding that retinoic acid and vitamin D can potentiate the other’s activity as VDR stimulation seems to be augmented in the presence of RAR:RXR binding [11].

### 2.3. Vitamin D Role in Immune Modulation

In addition to the role of vitamin D and the VDR in normal hematopoiesis and leukocyte differentiation the presence of the VDR on activated lymphocytes suggests a role in immune modulation on differentiated cells. VDR knock-out (KO) mice have a normal number of T and B lymphocytes but there are changes in the cytokine profile in VDR knock-out mice that affect the T-helper (Th) immune response. Inflammatory cytokines can elicit a cellular immune response termed “Th1” or a humoral immune response termed “Th2”. The Th1 response is stimulated by IL2 and IFN-γ as major mediators, while the Th2 response is driven by IL-4, IL-6, and IL-10 [9]. In general, stimulation of the VDR receptor has been shown to favor the Th2 response by suppressing IFN-γ and this underlies the immune-modulatory effects of Vitamin D [8,9]. This is somewhat confounded, however, by the fact that in the context of complete vitamin D knock-out, the Th1 response is actually impaired, possibly through inhibition of IL-18 [9]. The VDR also seems to be crucial for proper development of invariant natural killer (iNK) cells, a subset of lymphoid cells involved in the most basic immune responses and also in restricting autoimmunity [15,16]. This was noted in the rodent VDR KO model in which iNKT cells were reduced. Reduced iNKT may promote autoimmunity but also blunted airway response to inflammation [15,16]. VDR receptor may also have a role in the homing of lymphoid cells to specific tissues and in attenuating inflammation. This has been shown by Yu and colleagues who studied a mouse model of inflammatory bowel disease. They were able to show that VDR KO animals developed an autoimmune inflammatory bowel disease in response to commensal flora, but the effect could be blunted with wild type CD4 cells [17]. Ultimately, they demonstrated that intraepithelial lymphocytes (with a pattern of CD4αα+) were dramatically reduced in VDR KO mice due to lower production and impaired tissue homing. CD4αα+ T lymphocytes are T-regulatory cells that help suppress inflammation by secretion of IL-10 and thus their reduced presence led to a fulminant colitis [17]. This provides an example of vitamin D mediated attenuation of an immune response suggesting that vitamin D may have a role in immune tolerance, the process by which autoimmunity is prevented.

## 3. Utilizing Vitamin D in Antineoplastic Therapy

### 3.1. Vitamin D as Differentiation Therapy for Myeloid Malignancies

*In vitro* studies show that vitamin D promotes differentiation of normal hematopoietic precursors and malignant myeloblasts, which has led to significant interest in studying vitamin D analogues as treatment for myeloid malignancies, particularly myelodysplastic syndrome (MDS) and acute myeloid leukemia (AML). MDS is a hematologic condition that typically manifests as cytopenias (low numbers of normal blood cells) and is due to acquired mutations in the DNA of hematopoietic stem cells causing difficulties in differentiation and production of mature cell types. Some forms of MDS may cause increased numbers of myeloblasts or “blasts,” which are immature, undifferentiated precursors of blood cells found circulating in the blood or present in the marrow and indicative of flawed differentiation of hematopoietic stem cells. These forms of MDS are considered higher risk as they are more likely to accumulate additional mutations that may lead to AML. The blast percentage, severity of cytopenias, age, and cytogenetics (chromosomal abnormalities found in tissue culture) are also used to risk stratify patients with MDS using tools such as the International Prognostic Scoring System (IPSS). AML is an aggressive blood cancer defined by replacement of normal hematopoietic cells with rapidly replicating myeloblasts, which are unable to undergo differentiation and thus accumulate in the marrow and peripheral blood. It may evolve spontaneously or as a secondary process from MDS. Much of the early research into therapy with vitamin D analogues paralleled the discovery that all-trans retinoic acid (ATRA), a ligand for RXR, could produce deep, durable remissions for acute promyelocytic leukemia (APML), a particular form of AML. This discovery supported the idea of differentiation therapy as a viable and exciting treatment for myeloid malignancies and an alternative to the classic approach using cytotoxic chemotherapy. Specific pre-clinical experience with HL-60 and other leukemic lines, such as U-937 and THP-1 have all shown differentiation and apoptosis of neoplastic myeloblasts with vitamin D, which would seem to suggest these compounds can, like ATRA in APML, convert differentiation-arrested myeloblasts into a mature blood cell [1,11,18]. The exact mechanisms by which this change is induced by Vitamin D receptor activation is not fully understood and investigations have revealed complex cross-signaling involving PI3 Kinase, activation of multiple pro-differentiation steps in the MAPK pathway, and likely upregulation of pro-apoptotic factors such as p53 [1]. Of note, early preclinical trials used supraphysiological doses of vitamin D to induce differentiation, raising concern therapeutic doses would necessarily cause hypercalcemia. [11,19] Early efforts, however, showed that fractionated dosing seemed to offer some promise for achieving differentiation with lower doses within physiological ranges [12].

### 3.2. Vitamin D as Therapy for Myelodysplastic Syndrome

There are seven studies of single agent vitamin D therapy for MDS. Koeffler and colleagues performed the first study of such a regimen in 1985, when they reported on 18 patients with MDS treated with single agent 1,25-dihydroxycholecalciferol up to a dose of 2 µg per day [19]. Although 8/18 patients had minor hematologic responses, no response persisted over the full 12 weeks of the study and hypercalcemia was a common toxicity of treatment [19]. It is important to note that this was a population at high-risk for death or the development of AML. Seven individuals had disease that transformed to AML over the study period [19]. Takahashi and colleagues treated 11 patients with MDS with 1-hydroxycholecalciferol and similarly saw partial or minor and transient responses in five of the 11 [20]. They were able to minimize hypercalcemia with intermittent dosing of the vitamin D, but this may have reduced efficacy [20]. Other studies of vitamin D analogues alone for MDS include Molnar and colleagues, who in 2007 reported treating 23 patients with low or intermediate risk MDS with 2000–4000 international units of cholecalciferol daily. They failed to show any hypercalcemia, but there were no hematologic responses [21]. Mellibovsky and colleagues reported a positive single arm study of 19 patients with low or intermediate risk MDS by IPSS treated with either 25-hydroxycholecalciferol (five patients) or 1,25 dihydroxycholecalciferol (14 patients) of whom 11/19 had significant hematologic response [22]. Of note, there were no cases of hypercalcemia in this study.

There are two studies looking at vitamin D2 analogues for MDS. Koeffler and colleagues treated 12 patients with low to high risk disease with a vitamin D2 analogue (Paracalcitol) with no responses noted; one patient had a doubling of his platelet count but succumbed to fatal fungal infection shortly afterward [23]. In 2008 Petrich and colleagues reported a phase II study with 15 MDS patients, 12 with low risk disease, who were treated with doxercalciferol, another D2 analogue with possibly lower risk of hypercalcemia [24]. Nine of 14 patients completed the full planned 12 weeks of treatment with five stopping early due to progressive disease and one due to hypercalcemia [24]. Six patients had stable disease and ultimately eight progressed while on study leading to a conclusion that doxercalciferol had at best minimal activity. Notably, both patients with CMML experienced worsening monocytosis that reversed upon discontinuation of doxercalciferol, which may suggest an *in vivo* differentiation effect [24].

One notable study is a series of 30 patients treated by Motomura and colleagues in which patients were randomized to supportive care or 1-hydroxyvitamin D3 starting at 1 µg and titrated up to 4–6 µg if tolerated [25]. The two groups were matched for diagnoses and had a reasonable balance of higher and lower risk patients. The patients allotted to vitamin D3 received on average 17 months of therapy. Two patients developed hypercalcemia on the treatment arm and only one had a transient hematologic improvement with the rest failing to improve. Nevertheless, only one of the 15 patients on vitamin D analogue therapy and 7/15 on the control arm progressed to AML leading to a statistically significant benefit in survival in the treatment group [25].

There have been efforts to combine vitamin D with other differentiation and cytotoxic agents. Blazsek and colleagues reported a case in 1991 of a patient with MDS who responded to a combination of *cis*-retinoic acid and vitamin D therapy, leading to the hypothesis that this combination could be useful for non-APML myeloid disorders [26]. Siitonen and colleagues reported the results of a series of 19 patients with all types and risk categories of MDS treated with a combination of 13-*cis*-retinoic acid, 1,25-dihydroxycholecalciferol (1 µg daily), and valproic acid [27]. Valproic acid in this case was being used as a histone deacetylase (HDAC) inhibitor. Overall 3/19 patients had minor hematologic improvement, but toxicity, general linked to valproic acid (fatigue, liver function changes) and retinoids (skin problems), led 8/19 patients to be intolerant of the regimen [27]. The authors ultimately concluded that the addition of vitamin D did not seem to add significant synergistic activity to the valproic acid regimen already known to have modest activity.

One promising path is the use of growth factors with differentiating agents. Ferrero and colleagues reported in 2008 on a study of 63 patients with MDS, excluding high risk patients with high numbers of blasts, treated with a combination of recombinant human erythropoietin as well as *cis*-retinoic acid; 1,25 dihydroxycholecalciferol; and 6-thioguanine if blasts were present [28]. The purpose of this study was primarily to assess erythroid response and compare it to the rates seen with erythropoietin alone or the combination of the two differentiating agents and thioguanine alone, which was approximately 30% for each. Overall, there was an erythroid response rate of 60% overall, which was better in the low risk patients than those with excess blasts [28]. There was also data for improved survival for the low risk patients who had a response, with 93% of responders *vs.* 58% of nonresponders alive at three years [28]. Though presented as a positive study, the lack of erythropoietin dose standardization or control arm makes the benefits from the new agents difficult to appreciate. Toxicity was generally mild and primarily related to the retinoid agents. Surprisingly, there was little emphasis on the cardiovascular mortality rate of 8/63 patients, which could have been associated with the use of the erythroid stimulating agent [28].

Another group of studies has looked at combinations of vitamin D analogues as differentiating agents with cytotoxic chemotherapy. Ferrero and colleagues reported on experience treating 53 MDS patients with 13-*cis*-retinoic acid (20–40 mg/day) and 1,25-dihydroxycholecalciferol (1–1.5 µg/day) with or without intermittent thioguanine, a cytotoxic agent [29]. Thioguanine was utilized three out of every six weeks for subjects with a marrow blast count of >5% or those who failed to respond to the dual differentiating agent therapy [29]. Ultimately, 40/53 patients ended up receiving thioguanine. Overall response rate was 60% but rates of complete response (CR) were low with only two patients in the entire cohort achieving that outcome. Still, 50% of patients had a decline in transfusion requirement, which is a clinically relevant outcome and could significantly improve quality of life [29].

### 3.3. Vitamin D as Therapy for Acute Myeloid Leukemia

MDS, particularly in its lower risk forms, may be an indolent disease with a natural history extending over years. AML, on the other hand, tends to be fatal over weeks to a short number of months without effective treatment. Therefore, therapeutic plans that include only differentiating agents such as vitamin D have tended to be used only in patients with very treatment resistant disease or those at high risk for side effects of conventional cytotoxic chemotherapy. The only studies of vitamin D monotherapy in AML consists of a total of five patients treated in Japan in the mid 1980s of whom 4/5 had transient improvements in blasts and 1/5 had reported brief normalization of the bone marrow as reported by Irino, Takahashi, and Nakayama [20].

There are at least two series of elderly AML patients treated with low dose cytarabine regimens (subcutaneous in Europe and low dose IV in the US) combined with vitamin D analogue and another agent. Low dose cytarabine is a relatively well-tolerated cytotoxic regimen that had been the standard of care for elderly or unfit patients with AML. Slapak and colleagues in 1992 reported the use of cytarabine at continuous infusion (20 mg/m^2^/day) for 21 days with oral hydroxyurea (500 mg twice daily) starting the day preceding cytarabine and continuing for 22 days, and 1,25 dihydroxyvitamin D (0.5 µg twice daily) from the initiation of therapy until relapse or end of study [30]. Of the 29 patients (all older than 62 years) there was an overall response rate 79% of which 13 (45%) were in CR [30]. Toxicity was primarily hematologic with severe neutropenia and thrombocytopenia common, though this is essentially an unavoidable side effect of all effective therapies for AML. Only two patients developed hypercalcemia, which was asymptomatic and required no further treatment than holding the vitamin D analogue. Median remission duration was 9.8 months with overall survival of 12 and 14 months for non-responders and responders, respectively [30]. These results are considered at least not inferior to those seen with low dose cytarabine alone. Ferrero and colleagues in 2004 report a similar low dose cytarabine (8 mg/m^2^ subcutaneously twice daily) and 1,25-dihydroxyvitamin D (1 µg daily) regimen with 13 *cis*-retinoic acid (20–40 mg daily) and thioguanine (40 mg daily). Thioguanine and cytarabine were given for the first two to three weeks with 1,25-dihydroxyvitamin D3 and 13 *cis*-retinoic acid for a five week course. Those who responded received dual differentiating agent therapy continuously with either daily thioguanine, one out of every three weeks, or 6-mercaptopurine + cytarabine for two weeks out of every five or six [31]. Thirty total patients were treated (24 with AML and 6 with MDS) and using similar response criteria to the Slapak study, this regimen showed a 50% response rate with 8 having a CR (27%) and seven having a partial response (PR) [31]. Once again, toxicity was primarily related to cytopenias and is difficult to separate from disease effect. Median survival was 7.5 months for the entire treatment arm but significantly better at 16.5 months in responders [31]. Toxicity was primarily related to profound cytopenias with no hypercalcemia reported [31]. As in other studies with retinoic acid derivatives, dry mouth and lips were a common side effect.

The only study of vitamin D analogue combined with chemotherapy with a control group in the published literature is that reported by Hellstrom and colleagues in Sweden [32]. Seventy-eight total patients were treated, 68 with MDS and 15 with AML (either proceeding from MDS or with blast count of <30%). Cytarabine was given in a divided dose of 15 mg/m^2^/day subcutaneously and continued until bone marrow cellularity was ≤50% of that at study initiation or until “unacceptable” peripheral cytopenias developed. At that point cytarabine was held to allow counts to stabilize then reintroduced unless there was failure for count recovery, clear signs of progression, intolerance, or response. Half of the patients received dual differentiating agent therapy in addition to cytarabine with 13 *cis*-retinoic acid at 1 mg/kg/day and 1α OH-vitamin D at a starting dose of 1 µg/day and increasing until mild hypercalcemia developed [32]. In this study there was no significant difference in response rate between the two arms with response rates of 26.1% overall for the study [32]. Median overall survival was 10.5 months and not different between the arms [32]. Rate of transformation to AML in the MDS cohort was similarly unchanged. Given increased toxicity in the experimental arm, this was felt to suggest no additive benefit of differentiation agents with low dose cytarabine in the management of high risk MDS/AML [32].

Unfortunately, the studies of vitamin D monotherapy for myeloid disorders suffer from limitations including lack of control group, variable response criteria, and unclear end-points, which makes drawing conclusions difficult. Ultimately, it seems a portion of patients with myeloid disorders may respond to vitamin D monotherapy but the numbers are likely small and the responses transient. Patients with lower grade disease and without blasts may have a higher likelihood of response. Combination therapies may show more promise but few of these include a control group and the use of other active agents makes it difficult to determine the activity of the vitamin analogues. The only vitamin D and chemotherapy combination regimen studied in a controlled trial failed to show benefit from the addition of vitamin D. It is possible that hypercalcemia *in vivo*, which was seen in almost all studies, limits the maximally tolerated dose, significantly diminishing the therapeutic potential of vitamin D based therapies. Further studies are needed to define a role of vitamin D therapy in the treatment of myeloid malignancies.

### 3.4. Vitamin D as Therapy for Non-Myeloid Hematologic Cancers

There are few studies examining the role of vitamin D in non-myeloid blood disorders. Multiple pre-clinical studies have shown activity of the vitamin D analogue EB1089 in the myeloma H929 cell line [33,34,35]. In this case, the agent appears to promote apoptosis, and induce cell cycle arrest by down regulation of cyclin dependent kinases, an activity that is augmented by transforming growth factor beta (TGF-β) [33]. There are pre-clinical studies demonstrating that vitamin D has an inhibitory effect on lymphoid neoplastic cells, but to date, no studies in humans have been performed [8].

## 4. Vitamin D as a Modulatory of Immune Response in Allogeneic Transplant

There is considerable interest in vitamin D analogues for their immune-modulatory effects. The last decade has brought a renaissance of immune therapies in oncology with the approval of rituximab, a humanized IgG1 targeting CD20 on lymphoma cells; ipalimumab, a CTLA-4 inhibitor that upregulates the immune response against melanoma; and promising results with PD-1 inhibitors, which also increase immune responses against many other malignancies. Hematologic malignancies have long been known to be susceptible to immune surveillance and allogeneic hematopoietic stem cell transplantation (HSCT) has been established as a curative therapy for many hematologic malignancies since the 1980s. HSCT is performed by replacing an individual’s entire hematopoietic system, and hence his or her immune system, with that of another person. HSCT is the only potential curative therapy for several hematological disorders but presents two major barriers associated with the immune system. To allow a donor immune system to persist without being rejected, the recipient must receive immune suppressive chemotherapy, which causes severe reduction in immune cells and a significantly increased risk of infection. Post-transplant, when the donor immune system has grown, it may attack or reject the recipient’s tissues in a process called graft *versus* host disease (GVHD). Treatment of GVHD requires immune suppressive medications, thus leading to a delicate interplay between the dangers of immune stimulation and immune suppression. Optimal therapy would involve stimulation of the donor immune system to react and destroy the blood disease, without driving the immune response to healthy recipient tissues or organs. This process of immune modulation is mediated by a complex system of lymphocytes and associated cytokine regulators and agents that can modulate this system are attractive to improve outcomes.

Vitamin D via the VDR has a role in immune regulation and vitamin D analogues are well-established therapies for some autoimmune conditions such as psoriasis. This effect is likely due to the known effects of vitamin D on activated T and B cells that may affect signaling, tissue targeting, or immune regulation. Of course, given their roles in monopoiesis and macrophage activity, VDR stimulation appears to be crucial to the activity of antigen presenting cells and this is felt to be responsible for the role VDR activity seems to play in response to infections. For example, in tuberculosis infection decreased vitamin D levels appear to increase the risk of tuberculosis disease and supplementation may help resolve infection [7,36]. The immunomodulatory role of vitamin D suggests possible importance in the outcome following HSCT. VDR genes are polymorphic in the human population and the genetic variation in VDR has been a subject of investigation in patients undergoing HSCT. These polymorphisms were discovered by variation in restriction enzyme cleavage sites and thus are defined and named for these enzymes (*i.e.*, *Taq*I, *Apa*I) with two different allele possibilities with different cleavage patterns. Though the exact ways in which various polymorphisms change vitamin D receptor activity are often unknown these genetic variations have been associated with variability in immune function and other vitamin D activities such as growth, bone formation, and susceptibility to infectious diseases [6].

Cho *et al*. reported an analysis of VDR polymorphisms in 147 Korean patients who underwent matched related donor HSCT. They analyzed recipient VDR polymorphisms and retrospectively evaluated the association with patient outcomes including infection, GVHD, overall survival (OS) and disease free survival (DFS) [37]. The most significant findings were a correlation between polymorphisms at the *Taq*1 cleavage site where heterozygotes, (those possessing at least one copy of the C allele), had better disease free survival (DFS) and overall survival than TT homozygotes [37]. The functional significance of this allelic variation is unknown and no direct link to high or low VDR activity is reported. This study also found that recipients having two copies of the “A” allele for the *Apa*I polymorphism experienced decreased rates of acute GVHD and infection [37]. Polymorphisms of *Apa*I have been correlated to VDR activity with homozygosity for the “a” allele associated with higher VDR activity [4].

Middleton and colleagues reported a cohort of 88 patients with myeloid malignancies whose VDR gene polymorphisms and those of their 80 sibling donors were analyzed and correlated to outcomes [6]. Like Cho and colleagues, they noted a marked trend toward diminished acute GVHD in recipients with the AA (low VDR activity) genotype. Donor results for *Apa*I polymorphisms were somewhat more confusing. Recipients of donors with high VDR activity genotype (aa) showed a trend toward more GVHD, though not statistically significant (*p =* 0.065), but recipients of donors with the low VDR activity genotype (AA) had a statistically significant increased rate of death (HR 2.027, *p =* 0.0232) [6]. This is somewhat unusual as GVHD is a major contributor of mortality from HSCT and typically increased rates of GVHD will lead to increased mortality. This decreased survival was not statistically significant in patients who received cyclosporine GVHD prophylaxis alone (*p =* 0.83) but was marked for those patients who received multiagent GVHD prophylaxis with cyclosporine and the addition of methotrexate (*n =* 17), ATG (*n =* 4), corticosteroids (*n =* 4), or multiple additional agents (*n =* 2) (HR > 12, *p* < 0.0.0001). Primary causes of death in this cohort were infection, relapse, and interstitial pneumonitis [6].

Finally, Bogunia-Kubik and colleagues published an analysis of 123 Polish patients with the inclusion of those receiving unrelated donors [4]. They found an association between the FF genotype, which is associated with high VDR activity, and outcomes following HSCT. If both the donor and recipient possessed the FF genotype, the recipient experienced higher risk of GVHD [4]. As in the other studies *Apa*I genotype also impacted risks of GVHD. Interestingly, different genotypes corresponded to higher GVHD risks depending on whether they were present in the donor or recipient. In this analysis, unlike the previous one by Middleton, donor AA (low VDR activity) genotype correlated with a higher risk for GVHD than donor genotypes with at least one “a” allele [4]. At the same time, recipient aa (high VDR activity) genotype had a higher risk of GVHD and death than the low VDR activity genotype, which is consistent with what has been seen in other studies [4].

Clearly, in its mediation of immune signaling the vitamin D receptor appears to have an impact on immune reconstitution after HSCT and subsequent risks of infection, graft *versus* host disease, and graft *versus* disease effect. This would suggest a wide range of therapeutic potential if these relationships can be better understood. Despite the fact that vitamin D through the VDR usually plays an immune modulatory role, it appears that the most vigorous phenotype of the vitamin D receptor, when present in the recipients of HSCT leads to worse GVHD and worse outcomes [4,6,37]. This is suggested to be due to a particular role of stimulating the immediate immune response and cytokine storm that initiates GVHD, which is likely through a paracrine effect with its presence in recipient tissue [4]. One could also see vigorous VDR activity as promoting antigen presentation and thus a vigorous immune response though there is less speculation on this mechanism. Regarding donor VDR genotype, results are contradictory. The idea of vigorous VDR signaling in donors being favorable (as seen in the study of Bogunia-Kubik and colleagues) could suggest VDR mediated immune activation could promote more aggressive immune surveillance of infection and preclinical disease relapse. In addition, the role of VDR in production and homing of T regulatory cells, such as the CD4αα+ cells seen in the rodent model, could lead to improved modulation of the reconstituted immune system and improve outcomes. This data is compelling in suggesting that VDR and likely vitamin D play a role in immune reconstitution and immune surveillance in transplant patients and the findings may lead to opportunities to improve outcome following HSCT by the use of vitamin D analogues. A better understanding of how vitamin D status may affect the immune milieu in the setting of different polymorphisms could be useful for making recommendations about vitamin D supplementation. Given the general recommendations to supplement vitamin D after transplantation, it may be important to study the role of supplementation on outcome particularly in recipients with vigorous VDR phenotypes. Such a relationship is suggested by unpublished data from Bogunia-Kubik and colleagues, which showed higher rates of GVHD in recipients with the high VDR activity (aa) genotype treated with supplementary vitamin D [4]. Similarly, these findings suggest that in some recipients of HSCT, supplementation could promote better immune function in the reconstituted immune system and help prevent infections or late forms of GVHD [38].

## 5. Vitamin D Status in Supportive Care for Treatment of Hematologic Malignancies

Finally, at the same time vitamin D status is examined as a factor in disease outcomes and the non-classical roles are explored, the classical role of vitamin D in regulating bone calcium deposition and bone density remains critical. Two populations in which significant attention has been paid to vitamin D status are patients after allogeneic stem cell transplant, who are known to have significant endocrinologic and nutritional risk factors for deficiency related to their treatment, and patients with multiple myeloma, a disease intimately related to bone health.

Pediatric allogeneic stem cell transplant recipients are at particular vulnerability to bone loss due to the high risks of total body irradiation and conditioning chemotherapy to cause endocrine failure syndromes prior to final adult growth and the deposition of primary bone mass in the 20s. Moustoufi-Moab and colleagues looked at 55 patients who received allogeneic transplant between the ages of 5–26 and who were at least three years from the procedure and compared their bone mineral density scores to healthy controls [39]. Eighty-nine percent of patients did have endocrinopathies, and post-transplant patients were found to have significantly lower bone mineral density than healthy controls. Rates of vitamin D deficiency were high but not significantly different between patients and controls in this analysis [39].

Studies of adults have been contradictory in regards to assessing vitamin D status of allogeneic stem cell transplant survivors, though timing at which assessment is done likely has a significant effect. Joseph and colleagues assessing patients on day 0 and day +100 found 70% and 58% of patients to be vitamin D deficient, respectively [40]. Of note, this was an observational study and the different time points were assessed in different cohorts of patients. Fourty-six of the 72 patients in the post-transplant cohort had bone mineral density measurements with high rates of osteopenia (83%) and osteoporosis (22%) but these values were not significantly different in the vitamin D deficient and non-vitamin D deficient patients [39]. Still, the small sample size makes this lack of difference difficult to confirm. Robien and colleagues looked at a mixed cohort of 95 adults and children after at least one-year post transplant and found a only 10% were frankly vitamin D deficient with an additional 24% being insufficient [41]. Of note, greater than 60% of the patients in the study were taking vitamin D supplementation and oral intake was the greatest factor related to adequate levels. A daily intake of 400–600 IU corresponded with adequate levels of serum vitamin D and, as anticipated, prednisone diminished serum vitamin D concentrations [41]. No analysis of bone mineral density was done in this study.

Given these findings, there are clear recommendations on management of vitamin D status and bone health for patients after allogeneic stem cell transplant. UK guidelines recommend DEXA scanning for all patients expected to be on chronic (>3 months) of corticosteroid therapy as well as measurement for and correction of vitamin D deficiency [42]. ASBMT guidelines recommend DEXA for older women, all allogeneic stem cell transplant recipients, or those on chronic steroids or calcineurin inhibitors [43]. Both recommend assessment for vitamin D deficiency, and correction if present.

Myeloma is a plasma cell malignancy that causes lytic bone disease as one of its hallmarks. Given the importance of vitamin D in bone formation, the role of vitamin D supplementation in myeloma has gained some attention. Myeloma is known to promote osteoclastic over osteoblastic activity as a factor in causing lytic bone lesions. This activity can be reversed by one of our newer and best agents for myeloma, bortezomib. It has been shown that vitamin D significantly promotes the ability of bortezomib to inhibit osteoclast proliferation and heal bone lesions [44]. This would suggest an important role for supplementation, or at least adequate repletion, during treatment in order promote healing of bone disease.

At the same time the role of vitamin D has been examined clinically and observationally. Badros and colleagues surveyed 100 consecutive patients seen in University of Maryland cancer center with multiple myeloma and found 75% of patients were frankly deficient or insufficient [45]. They used this information to emphasize the need for checking and supplementation, particularly as bisphosphonates that are widely recommended in MM have never been shown to be effective without adequate calcium and vitamin D levels [45]. Additionally, attempts have been made to correlate vitamin D levels with myeloma behavior. Two observational studies from the Mayo clinic and Australia look at the clinical characteristics of vitamin D deficient myeloma patients [46,47]. Diamond and colleagues in New South Wales followed a group of myeloma patients for 10 years and stratified them into quartiles by serum vitamin D concentration. Statistically significant differences in paraprotein level and albumin concentration were seen with vitamin D quartile with lower quartiles associated with worse myeloma markers [47]. The lowest quartile also had significantly worse bone mineral density compared to the other quartiles. On the other hand, Ng and colleagues at the Mayo clinic looked at 148 newly diagnosed patients and were able to correlate 1,25-dihydroxyvitamin D levels of <50 nmol/L with lower albumin and higher CRP [46]. There was also a trend toward higher levels of vitamin D deficiency with higher staged myeloma, but there was no difference in skeletal events between the groups [46]. It should be noted that as observational studies both of these analyses cannot show causation, as it is impossible to know whether bad myeloma influences vitamin D status or vitamin D status influences myeloma behavior. Still, given the role of vitamin D in helping repair bone damage repletion is likely prudent to assure adequate repletion during treatment.

Ultimately, studies have revealed that post-allogeneic stem cell patients are vulnerable to endocrinopathies and bone mineral loss. Supplementation of fairly low doses seems to correspond to adequate levels and despite somewhat unclear direct relationships between vitamin D level and bone density at one point in time it seems prudent to maintain normal levels in patients particularly vulnerable to bone disease. Similarly, data that vitamin D is required for bisphosphonate activity and may help bortezomib, an important agent used for treatment of myeloma, suggests that those with lytic skeletal disease should at least receive replacement to assure a normal vitamin D level.

## 6. Summary and General Conclusions

Ultimately, the ubiquity of the vitamin D receptor and the myriad physiologic effects that have been found suggest multiple mechanisms of potential benefit from the use of Vitamin D in the treatment of hematologic disease. In the hematopoietic system there is evidence that the vitamin D pathway affects both differentiation of cells and their ultimate activation once differentiated, although the importance in various disease states remains poorly understood. It can be said with some level of certainty that vitamin D promotes differentiation of monocytes and macrophages under certain conditions, and there is a suggestion that at least a fraction of cases of myeloid disorders (*i.e.*, CMML) may respond to vitamin D supplementation. Similarly, immune-modulatory effects of vitamin D almost certainly affect the complicated immune environment in patients who have had allogeneic stem cell transplants. As the modulation of their immune system is a major factor in the clinical outcomes of these patients, understanding vitamin D signaling better in this situation may be helpful. Well-controlled trials will likely be necessary to confirm any anti-leukemic or anti-MDS benefits of vitamin D therapy. Continued scientific investigation of immune modulation and the role of vitamin D in that process is still necessary to understand proper mediation of immune function with vitamin D.
